# Lacking ARHGAP25 mitigates the symptoms of autoantibody-induced arthritis in mice

**DOI:** 10.3389/fimmu.2023.1182278

**Published:** 2023-05-10

**Authors:** Domonkos Czárán, Péter Sasvári, Ádám István Horváth, Krisztina Ella, Ágnes Réka Sűdy, Éva Borbély, Kitti Rusznák, Boldizsár Czéh, Attila Mócsai, Zsuzsanna Helyes, Roland Csépányi-Kömi

**Affiliations:** ^1^ Department of Physiology, Semmelweis University, Budapest, Hungary; ^2^ Department of Pharmacology and Pharmacotherapy, Medical School, University of Pécs, Pécs, Hungary; ^3^ National Laboratory for Drug Research and Development, Budapest, Hungary; ^4^ Histology and Light Microscopy Core Facility, Szentágothai Research Centre, University of Pécs, Pécs, Hungary; ^5^ Department of Laboratory Medicine, Medical School, University of Pécs, Pécs, Hungary; ^6^ Chronic Pain Research Group, Eötvös Loránd Research Network, University of Pécs, Pécs, Hungary; ^7^ PharmInVivo Ltd., Pécs, Hungary

## Abstract

**Objective:**

Despite intensive research on rheumatoid arthritis, the pathomechanism of the disease is still not fully understood and the treatment has not been completely resolved. Previously we demonstrated that the GTPase-activating protein, ARHGAP25 has a crucial role in the regulation of basic phagocyte functions. Here we investigate the role of ARHGAP25 in the complex inflammatory process of autoantibody-induced arthritis.

**Methods:**

Wild-type and ARHGAP25 deficient (KO) mice on a C57BL/6 background, as well as bone marrow chimeric mice, were treated i.p. with the K/BxN arthritogenic or control serum, and the severity of inflammation and pain-related behavior was measured. Histology was prepared, leukocyte infiltration, cytokine production, myeloperoxidase activity, and superoxide production were determined, and comprehensive western blot analysis was conducted.

**Results:**

In the absence of ARHGAP25, the severity of inflammation, joint destruction, and mechanical hyperalgesia significantly decreased, similarly to phagocyte infiltration, IL-1β, and MIP-2 levels in the tibiotarsal joint, whereas superoxide production or myeloperoxidase activity was unchanged. We observed a significantly mitigated phenotype in KO bone marrow chimeras as well. In addition, fibroblast-like synoviocytes showed comparable expression of ARHGAP25 to neutrophils. Significantly reduced ERK1/2, MAPK, and I-κB protein signals were detected in the arthritic KO mouse ankles.

**Conclusion:**

Our findings suggest that ARHGAP25 has a key role in the pathomechanism of autoantibody-induced arthritis in which it regulates inflammation *via* the I-κB/NF-κB/IL-1β axis with the involvement of both immune cells and fibroblast-like synoviocytes.

## 1 Introduction

Rheumatoid arthritis (RA) is a chronic autoimmune disease associated with inflammation and tissue damage and has a prevalence of nearly 2% (1). In its pathogenesis, a wide range of leukocytes is involved, e.g., lymphocytes, macrophages, osteoclasts, and neutrophils (1–3). Besides these cells, fibroblast-like synoviocytes (FLS) play an essential coordinating/regulatory role through their cytokines. In addition to osteoclast-mediated bone erosion, matrix metalloproteinases and cyclooxygenase-2 released from FLS cells are also involved in cartilage destruction. Recent studies reported the key role of NF-κB signaling in FLS responses (1, 2). Although several successful therapeutic approaches are available in RA, there is still a need for better targets (4).

Previously we discovered a new regulatory molecule of the Rho-family small G protein RAC, ARHGAP25, which, as a GTPase-activating protein (GAP) catalyzes the GTP hydrolysis of RAC, and this way, controls phagocytosis and superoxide production of neutrophilic granulocytes (5). We also revealed that ARHGAP25 is a key regulatory element of leukocyte transendothelial migration and is involved in the mobilization of hematopoietic stem cells and progenitor cells from bone marrow through CXCL12 (6). Thuault et al. provided the first evidence that ARHGAP25 regulates also non-hematopoietic cells by promoting the invasive potential of alveolar rhabdomyosarcoma cells through the regulation of RAC along the RHOE/ROCK/ARHGAP25/RAC axis (7). Recent studies confirmed that in pathological conditions decreased expression of ARHGAP25 resulted in increased migration and metastasis of different tumor cell types (8, 9). The fact that even the relatively weak expression of ARHGAP25 in non-hematopoietic cells (compared to immune cells) resulted in significant alterations in pathological conditions, raises the possibility that it has a key role in inflammatory diseases as well.

Since data are available only on the role of ARHGAP25 in neutrophilic granulocytes and B cells regarding immune cells (5, 10, 11), we investigated the role of ARHGAP25 in complex inflammatory conditions in the K/BxN serum transfer arthritis (STA) mouse model (12, 13). The K/BxN STA model is widely used to study the immunological responses characteristic of the effector phase of rheumatoid arthritis (3, 14–16). In this model, autoantibodies against the ubiquitously expressed glucose-6-phosphate isomerase are deposited into the ankle joints and stimulate neutrophilic granulocytes macrophages, mast cells, and osteoclasts *via* Fcγ receptors (12, 14, 17–19). Activation of phagocytes is followed by the release of reactive oxygen species and degrading enzymes, which lead to tissue damage (20), while osteoclasts are responsible for bone resorption (21). Mast cells and platelet-derived microvesicles are also involved in the development of arthritis by releasing proinflammatory cytokines (22, 23). Although hematopoietic cells are key elements of tissue damage in arthritic conditions, fibroblast-like synoviocytes are also essential for the development of the disease (24, 25).

Albeit the role of ARHGAP25 has been proven in regulating the functioning of phagocytes and osteoclasts (5, 10, 26), moreover, more and more studies are aiming to understand its role, as well as other GAPs in disease, little is known about their exact regulatory function in arthritis. In the present study, we show that 1) lacking ARHGAP25 mitigates the symptoms of serum-transfer arthritis in mice, 2) phagocyte infiltration into the inflamed ankle joint, but not the phagocyte effector functions, is reduced in ARHGAP25 knock-out animals, and 3) ARHGAP25 is expressed in fibroblast-like synoviocytes in an amount comparable to neutrophils. Our findings suggest that ARHGAP25 stimulates IL-1β production *via* the inhibition of NF-κB through I-κB.

## 2 Materials and methods

### 2.1 Animals

Age-matched male wild-type (WT) and *Arhgap25^-/-^* (KO RRID: MGI:5897665) mice on a C57BL/6 background were kept in a conventional animal facility in individually sterile ventilated cages (Tecniplast, Buguggiate, Italy). All experiments complied with the ARRIVE guidelines. Experiments of this study were carried out in accordance with the EU Directive 2010/63/EU for animal experiments act and were approved by the Animal Experimentation Review Board of Semmelweis University and the Government Office for Pest County (Hungary) (Ethical approval numbers: PE/EA/1967-2/2017, BA/73/00070-2/2020.).

For the generation of *Arhgap25^-/-^* bone marrow chimera mice, WT recipient animals - carrying the CD45.1 allele on the C57BL/6 genetic background - were lethally irradiated with 11 Gy from a 137Cs source using a GSM D1 irradiator as described previously (27). After that, unfractionated bone marrow cells from femurs and tibias of ARHGAP25^-/-^ mice (carrying the CD45.2 allele) were injected into the retroorbital plexus of recipient mice. In the case of mixed bone marrow chimeras, bone marrow cells from WT and *Arhgap25^-/-^* mice were equally mixed and injected into WT recipients. In reverse WT bone marrow chimeras, *Arhgap25^-/-^* mice were irradiated and the donor cells originated from WT mice carrying the CD45.1 allele.

Repopulation of the hematopoietic compartment by donor-derived cells was verified 4 weeks after the transplantation. Peripheral blood samples were collected and stained for Ly6G and CD45.1 or CD45.2. Samples were analyzed by flow cytometry (CytoFLEX, Beckman Coulter). The percentage of donor-derived cells among neutrophils was quantified. The median percentage of reconstitution of donor cells was 97.63%+2.99% in the case of ‘normal’ chimeras, and 98.65%+2.37% in the case of reverse chimeras. Animals were included in the study if the ratio of the donor cells was over 95% in the sample. For gating strategies, see **Supplementary Figure 1**. Bone marrow chimeras were used 4 weeks after the transplantation.

### 2.2 Autoimmune arthritis induction

Arthritic (K/BxN) and non-arthritic control (BxN) serum was obtained by crossing transgenic KRN^+/-^ (TCR) male with A^g7^ MHC II^+/+^ NOD/Lt female mice and collecting the serum of the F1 offsprings in the laboratory of Professor Attila Mócsai. In our experiments WT, *Arhgap25^-/-^*, or bone marrow chimeric mice were injected i.p. with 150 µl pooled K/BxN or BxN serum diluted in sterile Phosphate Buffered Saline (PBS) in 1 ml volume per mouse. Prior to treatment, and every day after for 8 days, the severity of arthritis was assessed in a blinded setup by scoring both hind limbs on a 0,5-10 scale based on the visible signs of inflammation (swelling, redness, and toe disfigurement), and by measuring ankle thickness with a spring-loaded caliper (Kroeplin) (15, 28).

### 2.3 Measurement of hind paw volume

Hind paw volume was measured using a plethysmometer (Ugile Basile) before and every day after arthritis induction, for 8 days. The measurement is performed by immersing the hind paw into a water-filled glass tube on the instrument. The plethysmometer contains a force transducer, which measures the displaced fluid volume, and according to this, calculates the paw volume expressed in cubic centimeters (cm^3^). During data analysis, the mean volumes of both hind paws were counted (28).

### 2.4 Measurement of joint function

Grasping ability was measured by horizontal grid tests 3, 6, and 8 days after arthritis induction. Mice were placed on a horizontal wire grid, then it was flipped over slowly and the time for which the animals were able to hold on to the grid was measured (maximum 20 seconds). At each time point, every mouse was measured three times and the average was used for evaluation using Keplen-Mayer survival analysis (15). Before serum treatment, mice were trained on the grid on three different days to correct the individual differences in grasping ability.

### 2.5 Measurement of mechanonociceceptive threshold

The mechanonociceptive thresholds of the hind paws were assessed using a dynamic plantar aesthesiometer (DPA, Ugo Basile), prior to and 3, 6, and 8 days after arthritis induction (29). Animals were placed individually into plexiglass boxes with wire grid bottoms where their movement was not restricted. Hind paws were touched from below with a metal filament with gradually increasing force (up to 10 g with 4 s latency) until avoidance movement occurred. Three measurements were performed on both hind paws, and their mean resulted in the mechanonociceptive threshold, which was expressed in gram (g).

### 2.6 Histology

Mice were terminally anesthetized *via* i.p. injection of 70 mg/kg sodium pentobarbital (Euthanimal, Alfasan Nederland B.V.) 8 days after arthritis induction. Ankles were harvested, fixed using 4% buffered formaldehyde, decalcified, dehydrated, and embedded in paraffin. Sections were prepared (5 µm) and stained with hematoxylin and eosin or Safranin O. Images were taken and evaluated by a trained observer blinded from the experiment. Synovial hyperplasia, the number of fibroblasts and collagen deposition, as well as cartilage destruction, were assessed and scored on a 0-3 scale, where 0 means the normal structure, 1 demonstrates mild, 2 moderate, and 3 severe changes (29)

### 2.7 *In vivo* imaging of neutrophil myeloperoxidase activity

The *in vivo* bioluminescence imaging of neutrophil-derived MPO activity was performed using IVIS Lumina III *In Vivo* Imaging System (PerkinElmer) (30, 31). One day before arthritis induction (day -1) mice were anesthetized with ketamine (Calypsol, Gedeon Richter Plc.; 120 mg/kg i.p.) and xylazine (Sedaxylan, Eurovet Animal Health B.V.; 6 mg/kg i.p.), and hair was totally removed from the hind paws and the ankle joints to avoid signal distortion. Prior to the measurements, (at 24 and 48 h, and on day 7) mice were anesthetized and Luminol sodium salt (Gold Biotechnology) dissolved in 1x PBS (30 mg/mL) was injected (150 mg/kg, i.p.). Bioluminescence intensity was assessed 10 min after luminol administration (120 s acquisition, Binning=8, F/Stop=1). To evaluate, identical regions of interest (ROIs) were applied around the hind paws of the animals, and luminescence was expressed as total radiance (total photon flux/s) within the ROIs.

### 2.8 *In vitro* extracellular superoxide production measurement of neutrophils

Bone marrow-derived neutrophils were isolated as described (10). Superoxide production was assessed upon three distinct stimuli. For measurement upon immune complex surface, 96 well microtiter plates (Nunc maxisorp) were coated with 20 µg/ml human serum albumin (HSA, Sigma Aldrich) in bicarbonate containing buffer (35 mM NaHCO_3_ and 15 mM Na_2_CO_3_) for one hour. After washing with Hank’s Balanced Salt Solution (HBSS, HyClone) HSA specific antibody (Sigma-Aldrich) was added in 1:200 dilutions in HBSS containing 10% (w/v) fetal bovine serum (FBS, Capricorn Scientific) for one hour. Integrin surface was created by coating the wells with 150 µg/ml fibrinogen (Calbiochem) for 30 minutes and after washing, adding mouse TNFα (Sigma-Aldrich) in 100 ng/ml final concentration. Phorbol 12-myristate 13-acetate (PMA, Sigma-Aldrich) was used in 100 nM concentration. In each case, 4x10^5^ neutrophils in HBSS containing 100 µM ferricytochrome c (Sigma-Aldrich) were added to the wells in three parallels (background superoxide production was also measured without stimulus). Change of OD (on 550 and 540 nm) was measured for one hour with an ELISA reader (ThermoFisher Scientific), and the amount of produced superoxide by 10^6^ cells was calculated.

### 2.9 Analysis of inflammatory infiltrate

Mice were sacrificed 5 days after serum treatment and ankle joints were flushed out with ice-cold PBS supplemented with 20 mM N-2-Hydroxyethylpiperazine-N’-2-Ethanesulfonic Acid (HEPES, Sigma-Aldrich) and 10 mM ethylenediaminetetraacetic acid (EDTA, Sigma-Aldrich). After centrifugation, supernatants were saved and concentrations of IL1-β and MIP-2 were measured with ELISA kits (R&D systems), using the protocol provided by the manufacturer. Sedimented cells were stained with PerCP-Cy5.5-conjugated anti-Ly6G and PE-conjugated F4/80 specific antibodies or isotype control (ThermoFisher Scientific) as described (10). Labeled cells were analyzed with a Cytoflex cytometer (Beckman Coulter).

### 2.10 Investigation of affected signaling pathways

Five days after arthritis induction, ankles were harvested, snap-frozen in liquid nitrogen, ground up, and lysed in lysing buffer (30 mM Na- HEPES, 100 mM NaCl, 1% [w/v] Triton- X-100, 20 mM NaF, 1 mM Na-EGTA, 1 mM Na-EDTA, 100 mM benzamidine, % [w/v] aprotinine, 1% [w/v] protease inhibitor cocktail, 1% [w/v] phosphatase inhibitor cocktail, and 1% [w/v] phenylmethylsulfonyl fluoride; pH 7.5). The protein concentration of the lysates was determined according to Bradford. Samples were separated on 4-15% polyacrylamide gradient gels (Bio-Rad) and were blotted to nitrocellulose membranes (Bio-Rad). After blocking with EveryBlot Blocking Buffer (Bio-Rad), membranes were incubated overnight at 4°C with the indicated phosphoprotein-specific primary antibodies (**Table 1**). Bound antibody was detected with enhanced chemiluminescence using horseradish peroxidase-conjugated rabbit IgG-specific secondary antibody (GE Healthcare) in 1:5000 dilution for one hour. Following the development to x-ray films (Fujifilm), membranes for phosphorylated ERK, MAPK, AKT, or GSK3β were stripped using 2% Sodium dodecyl sulfate (SDS, Sigma-Aldrich) and 0,7% 2-mercaptoethanol (Serva) in PBS at 55°C for 20 minutes and decorated with the antibodies against total proteins (**Table 1**). After a second stripping step, GAPDH was labeled as a loading control (Cell Signaling antibody, 1:5000). In the case of I-κB, membranes were stripped only once to detect GAPDH. Membranes decorated with anti-E-cadherin or β-catenin were not necessary to strip, since their molecular weight is not overlapped with GAPDH, and we did not detect the phosphorylated forms. Densitometric analysis of x-ray films was carried out with ImageJ software (ver. 1.53o). For data normalization, we used GAPDH in the case of total protein content and the appropriate total protein in the case of phosphoproteins.

**Table 1:** Antibodies used for Western blot analysis.

Specificity	Dilution	Manufacturer	Catalog number	RRID
Total-p44/42 ERK 1/2	1:1000	Cell Signaling	4695S	AB_390779
Total-p38 MAPK	1:1000	Cell Signaling	9212S	AB_330713
Total-Akt	1:1000	Cell Signaling	4691S	AB_915783
Total-NF-κB p65	1:1000	Cell Signaling	8242S	AB_10859369
Total-GSK3β	1:1000	Cell Signaling	#9832	AB_10839406
Total-E-cadherin	1:1000	Cell Signaling	#3195	AB_2291471
Total-I-κB	1:1000	Cell Signaling	4812S	AB_10694416
Total-β-catenin	1:500	Cell Signaling	#8480	AB_11127855
Phospho- p44/42 ERK 1/2	1:1000	Cell Signaling	4370S	AB_2315112
Phospho-p38 MAPK	1:1000	Cell Signaling	4511S	AB_2139682
Phospho-Akt	1:1000	Cell Signaling	4060S	AB_2315049
Phospho-NF-κB p65	1:500	Cell Signaling	3033S	AB_331284
Phospho-GSK3β	1:1000	Cell Signaling	D85E12	AB_10013750
GAPDH	1:5000	Cell Signaling	14C10	AB_561053
ARHGAP25	1:5000	ImmunoGenes	–	–
Rabbit IgG (secondary, produced in goat)	1:5000	GE Healthcare	RPN4301	AB_2650489

### 2.11 Assessment of ARHGAP25 expression in rheumatoid arthritis synovial fibroblasts

Seven days after serum treatment all four ankle joints were excised, the skin was removed, and the ankles were washed with sterile 70% ethanol and then with HBSS. Joints were cut into little pieces in HBSS containing 20 mM HEPES and 80 mg Liberase enzyme cocktail (Roche) and incubated at 37°C for one hour with 1400 RPM shaking. After filtration with a 70 µm pore size filter (Greiner), samples were centrifuged, the pellets were washed twice with HEPES containing HBSS, and finally suspended in 10 ml Dulbecco’s modified Eagle medium with GlutaMAX I (Lonza) supplemented with 10% (w/v) FBS, 50 units/ml penicillin, and 50 μg/ml streptomycin. Cells were grown in a 5% humidified CO_2_ incubator at 37°C for one week on tissue culture Petri dishes (Orange Scientific). Cell lysates were Western blotted as described above and membranes were decorated with anti-ARHGAP25 and anti-GAPDH primary antibodies, and then with HRPO (horse-radish peroxidase) conjugated, rabbit IgG-specific secondary antibody (**Table 1**).

### 2.12 Statistical analysis

All data were analyzed and plotted using GraphPad Prism 8.0.1 Software. Comparison of experimental groups in the case of horizontal grid test was carried out by Kaplan-Meier survival analysis, followed by log-rank test. Histological analysis was performed with the Mann-Whitney test. In all other cases, two-way ANOVA with Tukey’s multiple comparison tests was used. All *p* values <.05 were considered statistically significant.

## 3 Results

### 3.1 Lacking ARHGAP25 significantly reduces the symptoms of K/BxN-induced arthritis

**Figure 1:**
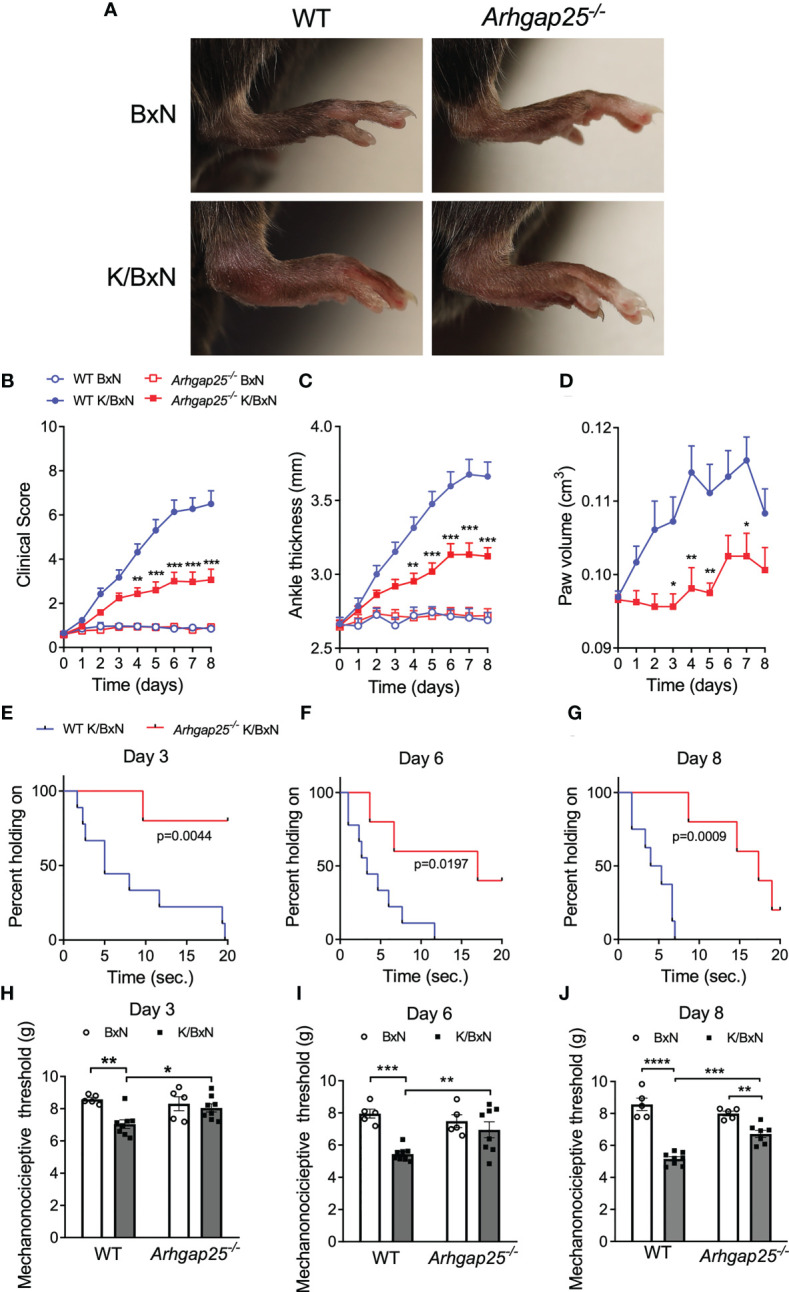
The absence of ARHGAP25 leads to a mitigated inflammatory phenotype in the K/BxN mouse model. WT and ARHGAP25-deficient animals were treated with either K/BxN (arthritogenic), or BxN (control) serum. Representative images of hind limbs of control or arthritic WT and Arhgap25^-/-^ mice **(A)**. Clinical scores, based on the visible signs of inflammation **(B)**, ankle thickness **(C)**, and hind paw volume **(D)** proved to be significantly lower in ARHGAP25 deficient animals compared to WT after arthritis induction. Mean +SEM of 21-23 (K/BxN) or 13-14 (BxN) animals in three independent experiments are plotted. The joint function was tested with a horizontal grid test and evaluated with Keplen-Mayer survival analysis. Lack of ARHGAP25 resulted in better retained articular function 3 **(E)**, 6 **(F)**, and 8 days **(G)** after arthritis induction. Eight or nine animals were analyzed per group. Mechanonociception was investigated using DPA. In ARHGAP25 KO mice, the mechanonociceptive threshold proved to be higher compared to WT, meaning that pain-related behavior upon mechanic stimulus is decreased in the absence of ARHGAP25 **(H–J)**. Mean ± SEM of 8 (K/BxN) or 5 (BxN) mice are plotted. *p < 0.05, **p < 0.01, ***p < 0.001, ****p < 0.0001.

To investigate the role of ARHGAP25 in a complex, inflammatory process, we used the K/BxN serum transfer arthritis murine model. Intraperitoneal injection of arthritogenic (K/BxN) serum resulted in a remarkable swelling of the hind paw of wild-type (WT) animals compared to control (BxN) serum-treated ones. However, in ARHGAP25 knock-out (KO) mice we only found a moderate change in hind paw morphology compared to control serum-treated KO. (**Figure 1A**). Scoring the severity of inflammation on a zero to ten scale and measuring the edema formation (ankle thickness and hind paw volume) revealed that lacking ARHGAP25 mitigated the symptoms of arthritis by about half (at days 3, 6, and 8, change in case of ankle thickening was 46%, 49%, and 53%, in the case of clinical score 35%, 56%, and 58%, and in the case of paw volume 100%, 63% and 64% respectively) compared to WT and this difference was significant (**Figures 1B–D**). To analyze the paw’s functionality, we performed a grid test, which yielded the results that ARHGAP25 KO mice spent a significantly longer time hanging on the wire grid compared to WT (p=0.0044, p=0.0197, p=0.0009 on days 3, 6 and 9 respectively, calculated with Kaplan Meier Survival Analysis and Log Rank test) (**Figures 1E–G**). Next, we wondered whether the mitigated articular function deterioration is associated with reduced pain-related behavior in KO mice. Thus, dynamic plantar aesthesiometry was carried out. Not surprisingly, arthritogenic serum treatment resulted in a significant reduction of the mechanonociceptive threshold in the WT animals compared to control serum-treated WT, and this effect became more pronounced over time. After K/BxN treatment, lacking ARHGAP25 significantly increased the mechanonociceptive threshold compared to WT and the threshold difference between BxN-treated and K/BxN-treated KOs became significant only on day 8, suggesting the reduced mechanonociception of the KO (**Figures 1H–J**). To reveal, whether the reduced pain-related behavior of the KO animals has a central component, or it is mainly peripheral, we stained endogenous ARHGAP25 in the histological sections of WT animals’ periaqueductal gray matter (PAG), the somatosensory cortex (SSC), and the spinal cord (SC) sections (**Supplementary Methods**, **Supplementary Figure 2**), and we performed Western blot analysis from the dorsal root ganglia (DRG) (data not shown). Although, we could observe specific staining in certain cells, probably in microglia, however, K/BxN treatment did not cause any change in the density of ARHGAP25+ cells compared to BxN treatment in the central nervous system. In DRG, we could not detect the protein. These data suggest that ARHGAP25 has mostly peripheral effects on pain-related behavior.

### 3.2 The absence of ARHGAP25 protects against cartilage destruction

**Figure 2:**
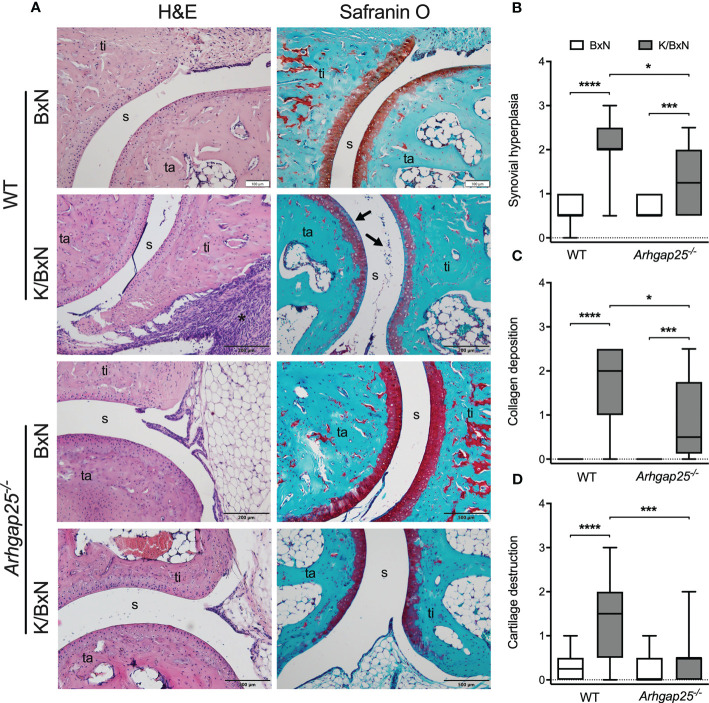
Histological analysis of ankle joints. Representative images of ankle sections stained with H&E or Safranin O (ta: tarsus, ti: tibia, s: synovium) **(A)**. Synovial hyperplasia is mostly caused by the hyperactive and rapidly proliferating fibroblast cells in WT arthritic ankles (A, asterisk). ARHGAP25 deficient animals are significantly less affected in this regard **(B)**. Fibroblast formation with collagen deposition can be observed in the case of arthritogenic serum-treated WT and to a significantly lesser degree in the case of *Arhgap25^-/-^* arthritic mice **(C)**. K/BxN serum treatment results in apparent cartilage destruction in WT (A, arrows) but not in the case of KO animals **(D)**. Box plots represent the semiquantitative histopathological scores of 16-19 (K/BxN) or 10-12 (BxN) animals. *p < 0.05, ***p < 0.001, ****p < 0.0001.

Eight days after the administration of arthritogenic or control serum, mice were sacrificed, and paw histological sections were prepared. Hematoxylin and eosin staining revealed extensive synovial hyperplasia in WT animals treated with arthritogenic serum, compared to control serum-treated WT. Parallelly, Safranin O staining showed considerable collagen deposition and cartilage destruction in WT animals suffering from arthritis (**Figure 2A**). Statistical evaluation of the pictures evinced that although synovial hyperplasia and collagen deposition were remarkably present in arthritogenic serum-treated KO mice, lacking ARHGAP25 reduced both parameters significantly compared to K/BxN serum-treated WT. In addition, cartilage destruction was not detectable in KO upon K/BxN serum administration in contrast to WT (**Figures 2B–D**).

### 3.3 Deletion of ARHGAP25 does not affect the MPO activity or superoxide production of neutrophils

**Figure 3:**
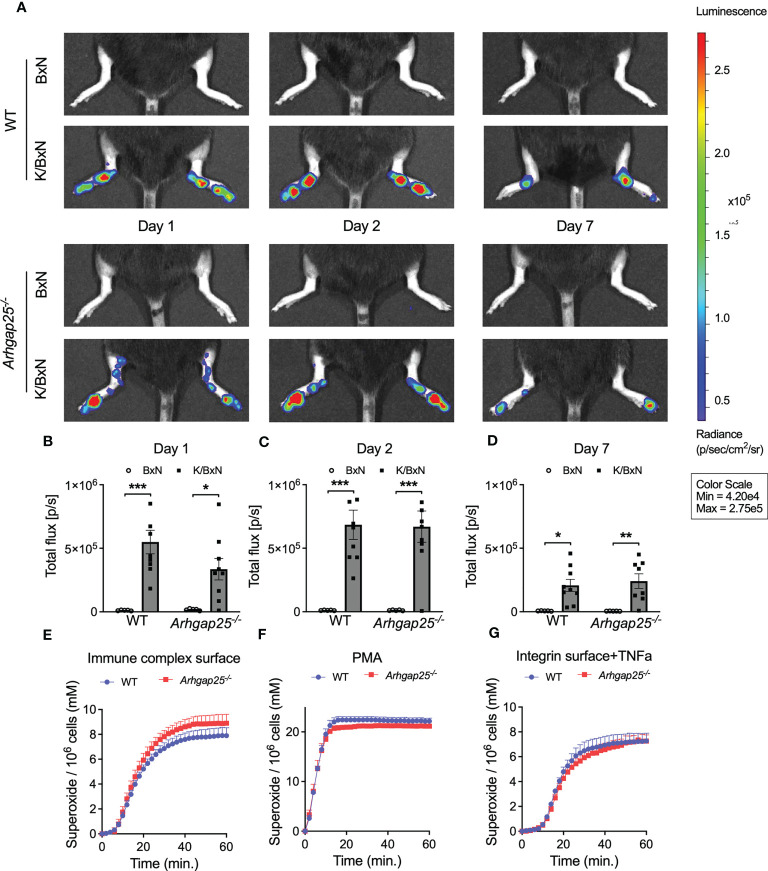
*In vivo* neutrophil MPO activity and *in vitro* neutrophil superoxide production. *In vivo* neutrophil MPO activity was tested with the IVIS Lumina III *In Vivo* Imaging System. One day after arthritis induction slightly reduced MPO activity could be observed in ARHGAP25-deficient mice **(A, B)** but after 2 or 7 days no difference between KO and WT was detectable **(A, C, D)**. Mean ± SEM of 9 (K/BxN) or 5 (BxN) mice are plotted. Extracellular superoxide production of bone marrow-derived neutrophils did not show any difference between KO and WT upon three different stimuli **(E–G)** Mean +SEM of 4-5 animals are shown. *p < 0.05, **p < 0.01, ***p < 0.001.

K/BxN serum transfer arthritis models the effector phase of the disease, in which neutrophilic granulocytes play a crucial role. Thus, we measured the myeloperoxidase (MPO) activity in the hind paw one, two, and seven days after arthritogenic serum administration *in vivo*. **Figure 3A** shows the representative pictures. Evaluating the total flux, which is proportional to the activity of MPO, we found that administration of the arthritogenic serum elevated the MPO activity to a similar extent in both groups. A slightly decreased MPO activity in KBxN-treated KO animals compared to arthritic WT was observed only on day one. (**Figures 3B–D**). Next, resting neutrophilic granulocytes were isolated from the bone marrow of non-treated WT and KO animals and superoxide production was measured upon different stimulations *in vitro*. Neither immune complex surface, PMA nor integrin surface supplemented with TNFα stimulation resulted in a difference in the superoxide production between KO and WT neutrophils (**Figures 3E–G**).

### 3.4 Leukocyte infiltration and inflammatory cytokine levels are decreased in absence of ARHGAP25

**Figure 4:**
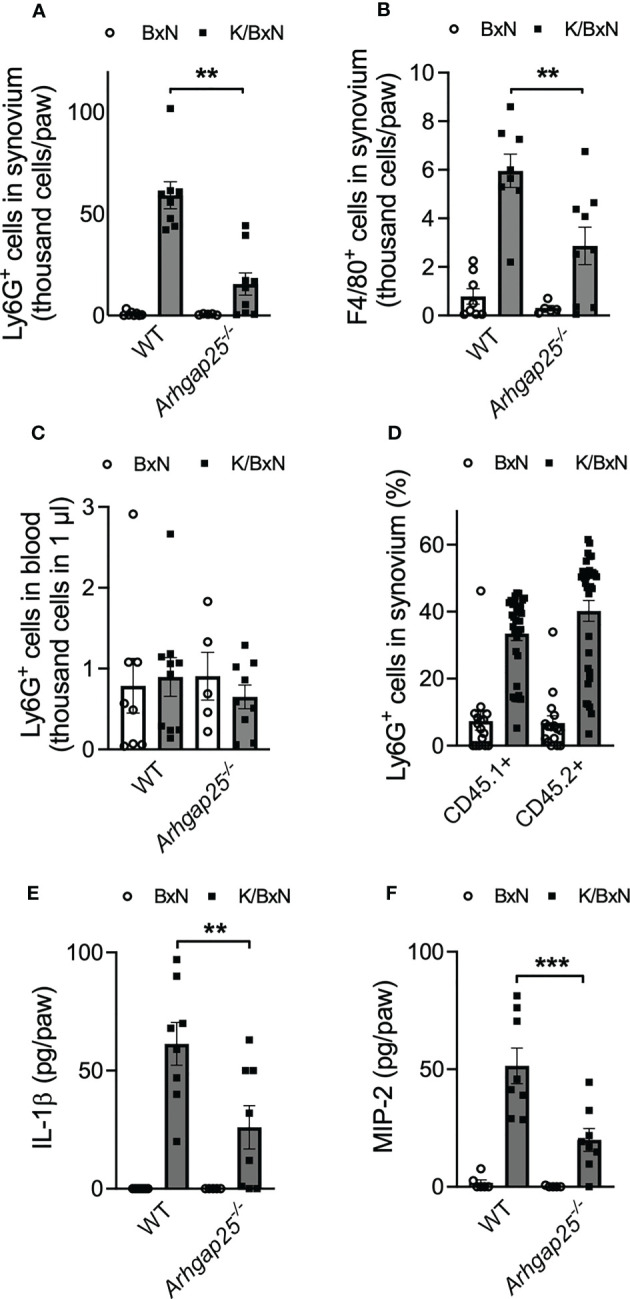
In the absence of ARHGAP25, the phagocyte count and the inflammatory cytokine content of the synovial infiltrate are reduced. Five days after arthritis induction, the synovium of mice was flushed out and analyzed *via* flow cytometry and ELISA. After K/BxN treatment the number of neutrophils was significantly lower in the synovium of *Arhgap25^-/-^* mice compared to WT **(A)**. Macrophages were also less abundant in the synovial infiltrate of K/BxN-treated KO animals **(B)**. Neutrophil counts were similar in KO and WT mice in the bloodstream **(C)**. Mean ± SEM of 9-10 (K/BxN) or 5-8 (BxN) animals are plotted. In the case of mixed bone marrow chimeras, with 50% WT (CD45.1+) and 50% ARHGAP25 deficient (CD45.2+) hematopoietic cells, the cell-autonomous migration into the synovium of KO neutrophils was slightly, but not significantly higher than of the WT neutrophils **(D)**. The graph represents the mean ± SEM of 32 (K/BxN) or 16 (BxN) mice. Lack of ARHGAP25 resulted in decreased amounts of two important inflammatory cytokines IL-1β **(E)** and MIP-2 **(F)**. Mean ± SEM of 8 (K/BxN) or 5-7 (BxN) animals are plotted. **p < 0.01, ***p < 0.001.

We analyzed the infiltration of neutrophilic granulocytes and macrophages into the inflamed ankle joint. Arthritogenic serum treatment resulted in a remarkable increase of Ly6G+ neutrophils and F4/80+ macrophages in the hind paw’s synovium of WT animals. The absence of ARHGAP25 reduced neutrophil infiltration by a third (**Figure 4A**) and halved macrophage infiltration compared to WT (**Figure 4B**). In the peripheral blood, no difference was observed in the Ly6G+ cell count independently from the genotype or treatment (**Figure 4C**). Next, mixed bone marrow chimeras were generated (in which the ratio of KO and WT hemopoietin cells is 1:1), to test the cell-autonomous migration of neutrophils under the same condition (27). We observed an increasing tendency in the infiltration of CD45.2-carrying KO cells upon arthritogenic serum treatment compared to CD45.1+ WT cells, although, the difference was not statistically significant (p=0.0757). After control serum treatment, no observable difference occurred between CD45.2+ KO and CD45.1+ WT cells (**Figure 4D**). The cytokine environment of the synovium may be relevant in the altered infiltration of neutrophils mentioned above. Thus, we investigated the possible changes in the concentration of two relevant cytokines: IL-1β is a well-known regulatory component of the inflammatory processes and MIP-2 is essential for leukocyte migration (3, 32, 33). The synovial concentration of both cytokines was elevated in WT upon K/BxN serum treatment, compared to control serum-treated WT while the lack of ARHGAP25 significantly reduced the elevation of both cytokines (**Figures 4E, F**).

### 3.5 ARHGAP25 regulates NF-κB signaling by preventing IκB degradation

**Figure 5:**
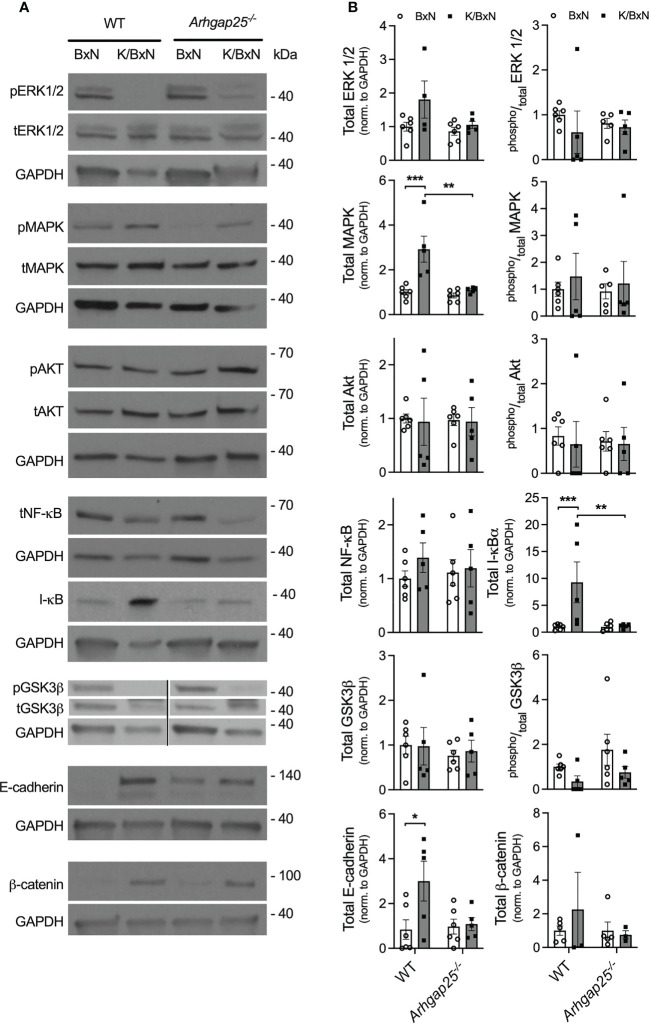
Analysis of the possibly affected signaling pathways in the K/BxN model. Five days after arthritis induction, lysates of snap-frozen ankles were prepared, separated on polyacrylamide gels, and comprehensive western-blot experiments were conducted. Representative western blots are shown for the MAPK/ERK, NF-κB, and WNT/β-catenin pathway **(A)**. Densitometric analysis of the Western blots **(B)**. Mean ± SEM of 4-6 mice are plotted. *p < 0.05, **p < 0.01, ***p < 0.001.

The above observations raised the question of which signaling pathway is responsible for the arthritis-mitigating effect of ARHGAP25. In Western blot experiments from total hind paw lysate, we investigated the expression of ERK1/2, MAPK, AKT, NF-κB, I-κB, GSK3β, E-cadherin, and β-catenin, as well as the phosphorylated forms of these proteins, where it was relevant (**Figure 5A**). Densitometrical analysis of the blots revealed a remarkable elevation of total I-κB in WT mice treated with arthritogenic serum, compared to control serum-treated WT, but this elevation was completely absent in arthritogenic serum-treated KO. Parallelly, total NF-κB showed an increased tendency in K/BxN-treated WT compared to KO, which suggests that ARHGAP25 plays a role in the regulation of NF-κB through I-κB. Similar alterations to I-κB were detected in the case of total E-cadherin and total MAPK. Total ERK1/2 changed in accordance with total MAPK, but only a tendency and no significant difference were found. In contrast, ARHGAP25 seemed not to be involved in the regulation of AKT, GSK3β, and β-catenin (**Figure 5B**).

### 3.6 ARHGAP25 expression in synovial fibroblast-like cells is comparable to that in neutrophils

**Figure 6:**
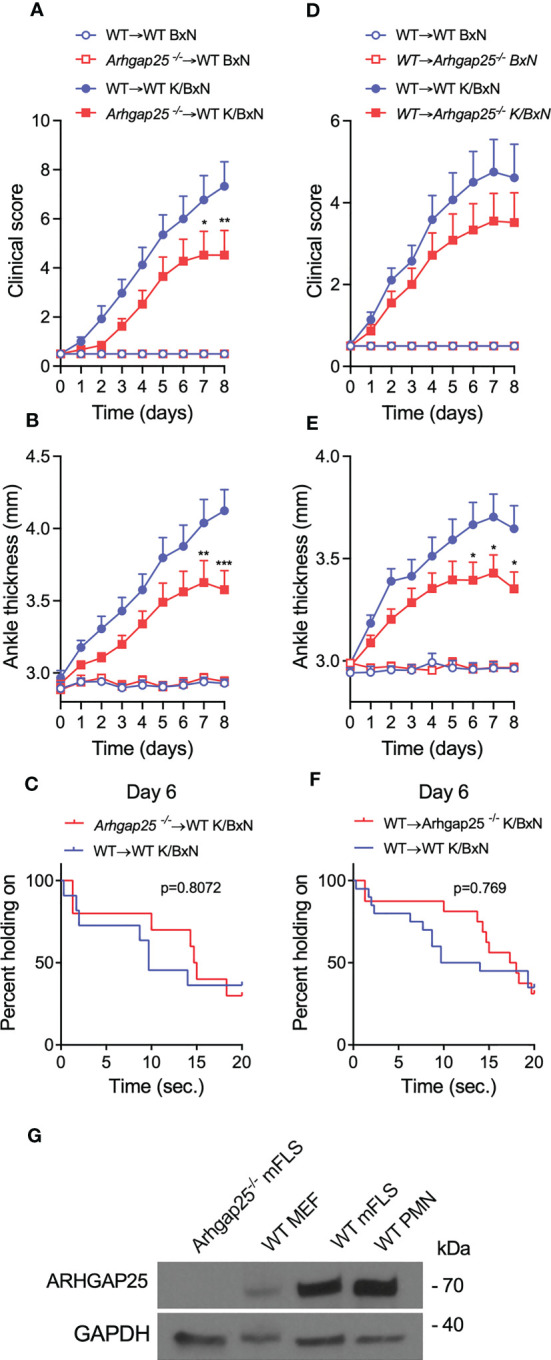
In bone marrow chimeric mice, the severity of inflammation was reduced slightly. Chimeric animals were created, which either express ARHGAP25 only in the hematopoietic compartment (WT→*Arhgap25^-/-^*, or reverse chimeras), or in every other cell, apart from hematopoietic cells (*Arhgap25^-/-^→* WT, or ‘normal’ chimeras). We evaluated clinical severity **(A, D)** and measured ankle thickness **(B, E)** after serum treatment. In the case of “normal” chimeras, 7-8 days after arthritis induction, significantly reduced clinical severity **(A)** and ankle thickness **(B)** were observed compared to WT chimeras. Mean +SEM of 10 (K/BxN) or 6 (BxN) animals are plotted in two independent experiments. In reverse KO chimeras, clinical score was mildly **(D)** and ankle thickening was significantly decreased after 6-8 days **(E)** Mean +SEM of 14-15 (K/BxN) or 6 (BxN) animals is shown in two independent experiments. To measure articular function we performed horizontal grid test and in both ‘normal’ and reverse chimeras, the articular function was slightly, but not significantly better retained in *Arhgap25^-/-^* chimeras **(C, F)**. Western blot was carried out from synovial fibroblast and neutrophil cell lysates. A representative picture shows high ARHGAP25 expression in RA fibroblast-like synoviocytes **(G)**. (mFLS: mouse fibroblast-like synoviocytes, MEF: mouse embryonic fibroblasts, PMN: Polymorphonuclear neutrophils.) *p < 0.05, **p < 0.01, ***p < 0.001.

To investigate whether ARHGAP25 plays a role in the effector phase of arthritis *via* other cell types besides hematopoietic cells, bone-marrow chimera and reverse chimera mice were generated. After administration of K/BxN or control serum, clinical score, ankle thickness, and grid test were performed. Interestingly, in the case of arthritogenic serum-treated “normal” chimeras carrying the KO hematopoietic compartment (*Arhgap25^-/-^* →WT), clinical score and ankle thickness showed only a slight decrease (55%, 31%, and 41% in the case of clinical score, and 41%, 30%, and 44% in the case of ankle thickening at days 3, 6, and 8, respectively) compared to the chimeras carrying the WT hematopoietic cells (WT→WT). However, from day 7, this decrease became significant (**Figures 6A, B**). We made a similar observation in the case of reverse chimeras except that we failed to detect any significant difference in the clinical score, and just a slight decrease for the benefit of reverse chimeras carrying the KO hematopoietic compartment (WT→*Arhgap25^-/-^*) (26%, 29%, and 26% at days 3, 6, and 8, respectively) (**Figure 6D**). However, ankle thickness differed significantly from day 6 compared to the reverse chimeras carrying the WT hematopoietic cells (WT→WT) (27%, 38%, and 42% at days 3, 6, and 8, respectively) (**Figure 6E**). Analyzing the articular functions, an increased tendency was detected in those cases, where the donor cells or the recipient animals were KO (*Arhgap25^-/-^* →WT and WT→*Arhgap25^-/-^*, p=0.88072 and p=0.769, respectively) (**Figures 6C, F**). These data suggest that ARHGAP25 is involved in the development of arthritis through the regulation of both hematopoietic and non-hematopoietic cells. This prompted us to investigate the expression of ARHGAP25 protein in fibroblast-like synoviocytes derived from arthritogenic serum-treated full WT animals. Surprisingly, an expression comparable to that found in neutrophils was detected, while in mouse embryonal fibroblast cells only a weak expression was observed. Fibroblast-like synoviocytes from KO mice treated with K/BxN serum were used as a negative control (**Figure 6G**).

## 4 Discussion

Previously it was believed that ARHGAP25 is a myeloid-specific GTPase activating protein (34), and is a key player only in the regulation of elementary phagocyte functions (5, 10). Recently more and more papers highlighted its expressional change and role in tumor behavior, especially in migration and metastasis (7–9, 35). These findings prompted us to question whether ARHGAP25 plays a role in complex inflammatory or autoimmune processes. To test this, we used the K/BxN serum-transfer arthritis murine model, which is a well-characterized model of autoantibody-induced tissue damage caused by the effector responses of mostly neutrophilic granulocytes and macrophages (12, 13).

Our present study proves that ARHGAP25 is a significant component of the development of autoantibody-induced arthritis. Its absence remarkably mitigated the phenotypical (e.g., ankle thickness, paw volume) and functional defects (e.g., articular function) of the disease. The milder symptoms in ARHGAP25-deficient mice can be explained by decreased joint destruction which is associated with reduced mechanonociception. Analyzing the periaqueductal gray matter, the somatosensory cortex, the spinal cord, and the dorsal root ganglia, we found ARHGAP25 expression only in some, we believe, microglial cells, which suggests that ARHGAP25 is involved in peripheral pain sensation, rather than central. However, how it participates in peripheral pain sensation is still an open question beyond the scope of this study.

Neutrophils and macrophages release several factors, e.g., reactive oxygen species and enzymes which under uncontrolled conditions can cause severe damage to host tissues (36). Surprisingly, the activity of neutrophil-derived myeloperoxidase and superoxide production - two well-known compounds –, in *Arhgap25^-/-^* animals did not show any alterations, which points in the direction that ARHGAP25-regulated elementary phagocyte immune responses cannot explain the reduced cartilage destruction and mitigated inflammatory phenotype in the knock-out. Previously we evinced that ARHGAP25 regulates leukocyte migration and many components of the process, but especially transendothelial migration is increased in the absence of the protein (10). Contrary to this, we found that neutrophil and macrophage infiltration into inflamed ankle joints was lower in the ARHGAP25-deficient mice compared to WT. Competitive migration assay revealed that our previously published observation is valid, and KO neutrophils have the capability of increased cell-autonomous migration, but this cannot manifest in this model. Based on this observation, along with the data from normal and reverse chimeras, we hypothesized that different factors originating from the non-hematopoietic compartment could be responsible for the lowered phagocyte infiltration in ARHGAP25 deficient animals. Pyrogen IL-1β is an essential mediator of inflammatory responses, as well as autoimmune inflammation, while the potent neutrophil-activator CXC chemokine MIP-2 recruits leukocytes to the site of inflammation (3, 37, 38).

Lacking ARHGAP25 significantly decreased the concentration of both cytokines compared to WT upon arthritis induction, which suggests that their lowered amount may lead to decreased phagocyte count and finally reduced cartilage destruction in the articular joint. We should note that through the activation of cyclooxygenase-2, IL-1β contributes to inflammatory pain hypersensitivity as well (39).

According to our observations, the total level of ERK1/2, MAPK, I-κB, and E-cadherin was increased in autoantibody-induced arthritis, in wild-type animals, and this increase was abolished in the knock-out. It was published that NF-κB inhibits IL-1β processing and secretion in macrophages and neutrophils, while IL-1β promotes the release of inflammatory mediators from fibroblast-like synoviocytes *via* the activation of NF-κB-ERK-STAT1 pathway (37, 40). We are aware of the limitations of our results since analysis of the nuclear translocation of NF-κB would be exact proof, which will be carried out within the framework of a further project. Nevertheless we hypothesize that ARHGAP25 prevents the degradation of I-κB leading to the inhibited nuclear translocation of NF-κB. This can stimulate the IL-1β secretion of macrophages and neutrophils. In absence of ARHGAP25 I-κB is degraded and releases NF-κB into the nucleus, resulting in a decreased IL-1β level. However, this contradicts what we found for MIP-2, as MIP-2 release is stimulated by NF-κB (38). The cooperation of fibroblast-like synoviocytes and CD4+ T cells promotes the release of a wide range of cytokines, e.g., IL-17a, IL-6, IFNγ, TNFα, as well as IL-1β, and all of these can stimulate MIP-2 release from macrophages (37). Moreover, other cells of hematopoietic origin (e.g., osteoclasts, mast cells, and platelets) are also involved in this model and all these cells express ARHGAP25, thus they can be partially responsible for the observed differences between WT and ARHGAP25 deficient animals [unpublished data, and (26, 41–43)]. However our hypothesis, together with our experiments carried out with chimeras and reverse chimeras suggests that beyond hematopoietic cells ARHGAP25 may play a regulatory role in fibroblast-like synoviocytes, and surprisingly, we found high expression of ARHGAP25 protein in these cells comparable to that found in neutrophils. We should note that even though the expression of ARHGAP25 in tumor cells is infinitesimal compared to neutrophils (unpublished data), it still has a significant role in the regulation of tumor invasion and metastasis (8, 9).

Taken together, our data indicate that ARHGAP25 is an important component of autoantibody-induced arthritis development, *via* the regulation of the cytokine environment. We suggest that ARHGAP25 regulates the macrophage/neutrophil/fibroblast-like synoviocyte cooperation through the modulation of I-κB – NF-κB signaling. Investigating the role of ARHGAP25 in this pathway by revealing its molecular interactions is a topic of further study.

## Data availability statement

The raw data supporting the conclusions of this article will be made available by the authors, without undue reservation.

## Ethics statement

The animal study was reviewed and approved by Animal Experimentation Review Board of Semmelweis University and the Government Office for Pest County, Hungary (Ethical approval numbers: PE/EA/1967-2/2017, BA/73/00070-2/2020).

## Author contributions

DC, PS: Formal analysis, Investigation, Writing-Original Draft. ÁH, ÉB, KR, KE, ÁS: Formal analysis, Investigation. AM: Resources. ZH, BC: Resources, Funding acquisition. RC-K: Conceptualization, Supervision, Writing-Original Draft, Funding acquisition. All authors contributed to the article and approved the submitted version.
