# Supplementary material for: Lacking ARHGAP25 mitigates the symptoms of autoantibody-induced arthritis in mice
Source: Front Immunol. 2023 May 10;14:1182278. doi: 10.3389/fimmu.2023.1182278 (PMC10208528; doi:10.3389/fimmu.2023.1182278)
Supplement: Supplementary file 1 [file DataSheet_1.pdf]

Supplementary Material

Lacking ARHGAP25 Mitigates the Symptoms of Autoantibody-induced Arthritis in Mice

Domonkos Czárán, Péter Sasvári, Ádám István Horváth, Krisztina Ella, Ágnes Réka Sűdy, Éva Borbély, Kitti Rusznák, Boldizsár Czéh, Attila Mócsai, Zsuzsanna Helyes, Roland Csépányi-Kömi*

***Corresponance:** Roland Csépányi-Kömi: csepanyi-komi.roland@med.semmelweis-univ.hu

1. Supplementary Methods: Immunohistochemistry from the Central Nervous System

For the immunostaining of somatosensory cortex (SSC), periaqueductal gray matter (PAG), and spinal cord (SC), free-floating sections were washed in PBS, treated with 3% H_2O_2 and blocked with 3% goat serum. After thorough washing with PBS, sections were incubated with monoclonal ARHGAP25 specific antibody overnight (1:1000, Abcam, ab192020) and then with anti-rabbit biotinylated secondary antibody (1:200; Vector Laboratories), washed and incubated in avidin-biotin-horseradish peroxidase (1:200; Vectastaine Elite ABC Kit, Vector). Labeled cells were visualized with 3,30-diaminobenzidine (Sigma-Aldrich) and H_2O_2 in PBS, then sections were mounted, dried, and dehydrated, cleared, and coverslipped with Eukit. Images were acquired and evaluated on a Nikon Eclipse Ti-U workstation.

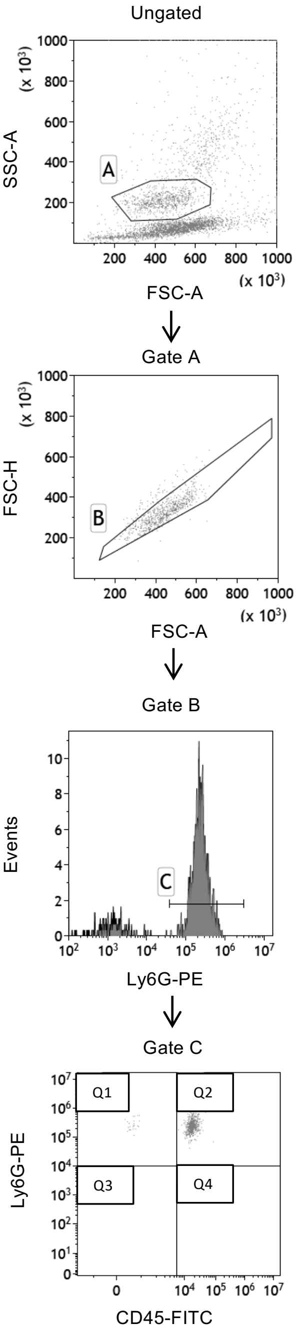
Ungated
SSC-A
(x 10³)
1000
800
600
400
200
A
200
400
600
800
1000
(x 10³)
FSC-A
Gate A
FSC-H
(x 10³)
1000
800
600
400
200
B
200
400
600
800
1000
(x 10³)
FSC-A
Gate B
Events
10
8
6
4
2
0
C
10²
10³
10⁴
10⁵
10⁶
10⁷
Ly6G-PE
Gate C
Ly6G-PE
10⁷
10⁶
10⁵
10⁴
10³
10²
10¹
0
Q1
Q2
Q3
Q4
0
10⁴
10⁵
10⁶
10⁷
CD45-FITC

Supplementary Figure 1. Flow cytometric analysis of the hematopoietic compartment in bone marrow chimera mice. Before analysis of specific labeling, neutrophils (A) and singlets were gated in all cases (B). Verification of the neutrophil population according to their Ly6G labeling (C). Donor cells were identified according to their Ly6G and CD45 labeling: in the case of *Arhgap25*$^{-/-}$ bone marrow chimera mice CD45.2-FITC antibody was used, and the ratio of double positive donor cells (Q2) was quantified. In the case of mixed bone marrow chimeras, CD45.2-FITC antibody was used and the Q1/Q2 cell ratio was measured. In reverse bone marrow chimeras, CD45.1-FITC antibody was applied and events in Q2 were identified as donor-derived cells.

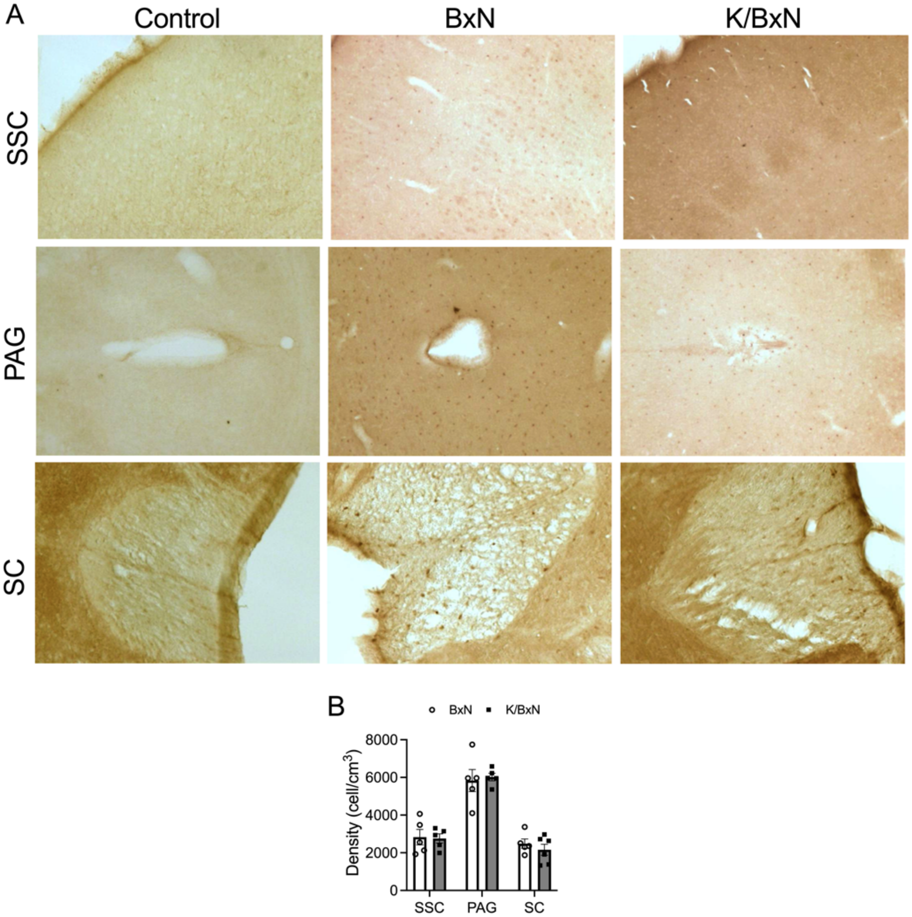
A
Control
BxN
K/BxN
SSC
PAG
SC
B
BxN
K/BxN
Density (cell/cm^3)
8000
6000
4000
2000
0
SSC
PAG
SC

Supplementary Figure 2. Immunohistochemistry of brain and spinal cord sections. Sections of the periaqueductal gray matter (PAG), the somatosensory cortex (SSC), and the spinal cord (SC) of WT animals were prepared and labeled for ARHGAP25 (control staining was also carried out in which case no primary, but only secondary antibody was used), images were taken (A) and labeled cells were counted (B). Although specific staining of the protein was detectable in certain cells, there was no difference between arthritic and control serum-treated sections in the density of ARHGAP25-expressing cells in the two brain areas or in the spinal cord (A, B). The mean ±SEM of 5 animals in each group were plotted.
